# Identification and characterization of Crumbs polarity complex proteins in *Caenorhabditis elegans*

**DOI:** 10.1016/j.jbc.2022.101786

**Published:** 2022-03-03

**Authors:** Victoria G. Castiglioni, João J. Ramalho, Jason R. Kroll, Riccardo Stucchi, Hanna van Beuzekom, Ruben Schmidt, Maarten Altelaar, Mike Boxem

**Affiliations:** 1 Division of Developmental Biology, Department of Biology, Faculty of Science, Institute of Biodynamics and Biocomplexity, Utrecht University, Utrecht, The Netherlands; 2 Division of Cell Biology, Neurobiology and Biophysics, Department of Biology, Faculty of Science, Institute of Biodynamics and Biocomplexity, Utrecht University, Utrecht, The Netherlands; 3 Division of Biomolecular Mass Spectrometry and Proteomics, Bijvoet Center for Biomolecular Research and Utrecht Institute for Pharmaceutical Sciences, Utrecht University, Utrecht, The Netherlands

**Keywords:** *C. elegans*, Crumbs, Crb, Stardust, PALS1, MAGU-2, PATJ, MPZ-1, cell polarity, epithelial polarity, FC, fold change, GUK, guanylate kinase, L27, Lin-2 and Lin-7 domain, NGM, Nematode Growth Medium, Nok, Nagie oko, PALS1, protein associated with Lin7, PATJ, PALS1-associated tight junction protein, PDZ, postsynaptic density 95, disks large, zona occludens-1, Sdt, Stardust, SH3, Src homology 3, Y2H, yeast two hybrid

## Abstract

Crumbs proteins are evolutionarily conserved transmembrane proteins with essential roles in promoting the formation of the apical domain in epithelial cells. The short intracellular tail of Crumbs proteins are known to interact with several proteins, including the scaffolding protein PALS1 (protein associated with LIN7, Stardust in *Drosophila*). PALS1 in turn binds to a second scaffolding protein PATJ (PALS1-associated tight junction protein) to form the core Crumbs/PALS1/PATJ complex. While essential roles in epithelial organization have been shown for Crumbs proteins in *Drosophila* and mammalian systems, the three *Caenorhabditis elegans crumbs* genes are dispensable for epithelial polarization and development. Here, we investigated the presence and function of PALS1 and PATJ orthologs in *C. elegans*. We identified MAGU-2 as the *C. elegans* ortholog of PALS1 and show that MAGU-2 interacts with all three Crumbs proteins and localizes to the apical membrane domain of intestinal epithelial cells in a Crumbs-dependent fashion. Similar to *crumbs* mutants, *magu-2* deletion showed no epithelial polarity defects. We also identified MPZ-1 as a candidate ortholog of PATJ based on the physical interaction with MAGU-2 and sequence similarity with PATJ proteins. However, MPZ-1 is not broadly expressed in epithelial tissues and, therefore, not likely a core component of the *C. elegans* Crumbs complex. Finally, we show overexpression of the Crumbs proteins EAT-20 or CRB-3 can lead to apical membrane expansion in the intestine. Our results shed light on the composition of the *C. elegans* Crumbs complex and indicate that the role of Crumbs proteins in promoting apical domain formation is conserved.

Cell polarity, the asymmetric distribution of components and functions in a cell, is a fundamental property of animal cells. In epithelial cells, proteins and lipids of the plasma membrane are distributed asymmetrically into an apical domain that faces the external environment or lumen and a basolateral domain contacting neighboring cells and the extracellular matrix. Cell–cell junctions at the boundary of the apical and basolateral domains seal the epithelial sheets to segregate the internal medium from the outside environment and provide mechanical strength to the tissue. The establishment of these different domains relies on mutually antagonistic interactions between evolutionarily conserved polarity complexes. The Scribble group proteins and the Par1 kinase promote basolateral identity, while the apical membrane domain is specified through the combined activities of the Par and Crumbs complexes (1, 2).

The composition of the Crumbs complex is dynamic and varies between tissues and developmental stage of the cell or organism. Nevertheless, the core configuration of the Crumbs complex is generally thought to contain the scaffolding proteins Crumbs, PALS1 (protein associated with Lin7—Stardust in *Drosophila*), and PATJ (PALS1-associated tight junction protein). All three proteins and the physical interactions between them are highly conserved from invertebrates to vertebrates. Crumbs is a transmembrane protein composed of a large extracellular domain, a transmembrane domain, and a short cytoplasmic tail that mediates interactions with other complex members through two protein interaction motifs: a FERM (band 4.1/Ezrin/Radixin/Moesin) domain-binding motif and a C-terminal PDZ (postsynaptic density 95/disks large/zona occludens-1) domain-binding motif (PDZ-binding motif). Crumbs was identified in *Drosophila* as an essential regulator of polarity in embryonic epithelia, follicular epithelial cells, and photoreceptor cells (3, 4, 5, 6, 7). In addition, Crumbs contributes to the control of tissue growth (8, 9, 10). Whereas *Drosophila* encodes one Crumbs protein, mammals express three Crumbs orthologs (Crb1–3). Crb1 and Crb2 are similar in size and domain composition to *Drosophila* Crumbs, while Crb3 lacks most of the extracellular region. Crb1 is predominantly expressed in retinal cells and loss or mutation of Crb1 causes various retinal defects in human and mice, including Leber congenital amaurosis and retinitis pigmentosa (11, 12, 13, 14). Human *CRB2* is expressed in the eye, brain, and kidney, while mouse *Crb2* is also broadly expressed during early embryonic development (15, 16). Knockout of *Crb2* in mice results in embryonic lethality during late gastrulation, likely due to disrupted polarity of epiblast cells, and conditional knockout of *Crb2* in the retina causes retinal degeneration defects similar to those observed in retinitis pigmentosa (13, 16, 17). Studies in zebrafish and mice also indicate an important role for Crb2 in podocyte development (18, 19). Finally, the *Crb3* gene is broadly expressed in most mammalian epithelial tissues (20, 21, 22). Knockdown and overexpression studies of CRB3 in MDCK cells, frog blastomeres, and human mammary cells indicate in important role for CRB3 in epithelial polarity establishment and junction formation (21, 23, 24, 25, 26). Crb3 KO mice die shortly after birth from epithelial defects such as cystic kidneys and abnormal intestine with apical membrane blebs and disrupted microvilli (26).

The intracellular domain of Crumbs interacts with PALS1, a member of the membrane-associated guanylate kinase family of adapter proteins. PALS1 contains a pair of Lin-2 and Lin-7 (L27) domains, a PDZ domain, a Src homology 3 (SH3) domain, and a guanylate kinase (GUK) domain (27). PALS1 interacts with the PDZ domain-binding motif of Crumbs through its PDZ and SH3 domains, additionally strengthening the interaction through its GUK domain (28, 29). PALS1 was identified in *Drosophila* as Stardust (Sdt) (30). In most *Drosophila* epithelial cells, Crumbs and Sdt are mutually dependent for their localization and loss-of-function mutations in either gene causing largely overlapping phenotypes (28, 29, 31, 32, 33, 34). The genetic interactions and interdependencies between Crumbs and Sdt are conserved in vertebrates. In zebrafish neuroepithelial cells, the expression pattern of the single PALS1 ortholog Nagie oko (Nok) resembles the combined pattern of three Crumbs family members, and Nok and Crumbs protein localization are mutually dependent (35). Apical localization of Nok in the myocardium and neural tube is also dependent on Crumbs2a, and Nok mutants that are unable to interact with Crumbs proteins recapitulate the phenotype of Nok loss-of-function mutants (36). In mice, conditional knockout of Pals1 in retinal progenitor cells disrupts the localization of Crumbs and Patj and results in a complex retinal phenotype that shares aspects of both *Crb1* and *Crb2* KO phenotypes, consistent with the involvement of Pals1 in Crb1 and Crb2 complexes (37, 38).

The final scaffolding protein of the Crumbs complex is PATJ, a protein characterized by the presence of an N-terminal L27 domain followed by multiple PDZ domains (four in *Drosophila* Patj and ten in mammalian PATJ). PATJ and PALS1 form an L27 tetramer through the interaction between two units of PALS1-L27N—PATJ-L27 (39, 40). The importance of PATJ for apical–basal polarization is less clear than for Crumbs and PALS1. Mammalian PATJ interacts with tight junction proteins including ZO-3 and claudin-1. Loss of PATJ causes defects in tight junction formation as well as a loss of Crb3 and PALS1 from tight junctions in cultured epithelial cells (41, 42, 43, 44, 45). In MDCKII cells, loss of PATJ has also been shown to cause defects in polarization and directional migration (43, 44). However, knockout studies of PATJ in vertebrates have not yet been reported. In *Drosophila*, PATJ is dispensable for the polarization of most epithelia and instead appears to play a more restricted role in regulating apical–basal polarity and Crb expression in photoreceptor cells and the follicular epithelium (46, 47, 48).

In mammals, a second structural homolog of PATJ exists, called MUPP1 (multi-PDZ domain protein 1), that consists of an N-terminal L27 domain followed by 13 PDZ domains (49). PATJ and MUPP1 have similar subcellular localization patterns and share several interaction partners, including PALS1 and the junctional proteins JAM1, ZO-3, and claudin-1 (50, 51). It is unclear, however, if MUPP1 is a regulator of polarity. In contrast to PATJ, knockdown of MUPP1 did not affect junction formation and polarization of mouse mammary epithelial cells, nor the localization of PALS1 (50). Nevertheless, in mouse retinal cells, MUPP1 coimmunoprecipitates with PATJ and shRNA gene silencing of *Pals1* in cultured mouse retinas or *in vivo* indicates that PALS1 is required for the localization of MUPP1 (13, 14, 38).

The subcellular localization of the Crumbs proteins in *Caenorhabditis elegans* is strikingly similar to that of Crumbs proteins in mammalian systems and *Drosophila* (52, 53). CRB-1 can provide a positional cue for junction formation in the intestine of animals depleted of both HMP-1 α-catenin and LET-413 Scribble (54). However, *C. elegans* Crumbs proteins are not essential for epithelial development in *C. elegans*, and even triple Crumbs deletion mutants are viable without obvious defects in cell polarity (52, 53, 54). Thus, the precise role of the Crumbs complex in *C. elegans* remains elusive. In addition, while putative orthologs of PALS1 and PATJ exist, it is not known whether they interact with each other or with any of the Crumbs proteins.

Here, we investigate the composition of the Crumbs complex and its role in apical polarity regulation in *C. elegans*. We demonstrate that the putative PALS1 homolog MAGU-2 interacts with all three Crumbs proteins and localizes to the apical membrane domain of epithelial cells in a Crumbs-dependent manner. This strongly suggests that MAGU-2 is the *C. elegans* ortholog of PALS1 and a member of the Crumbs complex. Like triple *crumbs* deletion mutants, animals lacking *magu-2* are viable and show no overt polarity defects. We also identify MPZ-1 as a candidate ortholog of PATJ, based on sequence similarity and the presence of a physical interaction with MAGU-2. However, expression of an endogenous MPZ-1::GFP fusion was limited to the nervous system, spermatheca, and excretory canal. MPZ-1 is therefore not likely to be a core component of the epithelial *C. elegans* Crumbs complex and may instead be a tissue-specific member with more specific functions. Finally, we show that overexpression of EAT-20 and CRB-3 in *C. elegans* resulted in apical membrane expansion, as reported for overexpression of *Drosophila* Crumbs. This effect was independent of the presence of MAGU-2, raising the possibility that MAGU-2 is not essential for the apical domain specifying activity of Crumbs in *C. elegans*. Together, our results indicate that the composition of the Crumbs complex and the role of Crumbs proteins in apical domain formation are conserved in *C. elegans*.

## Results

### MAGU-2 is the *C. elegans* ortholog of PALS1 and interacts with Crumbs proteins

**Figure 1:**
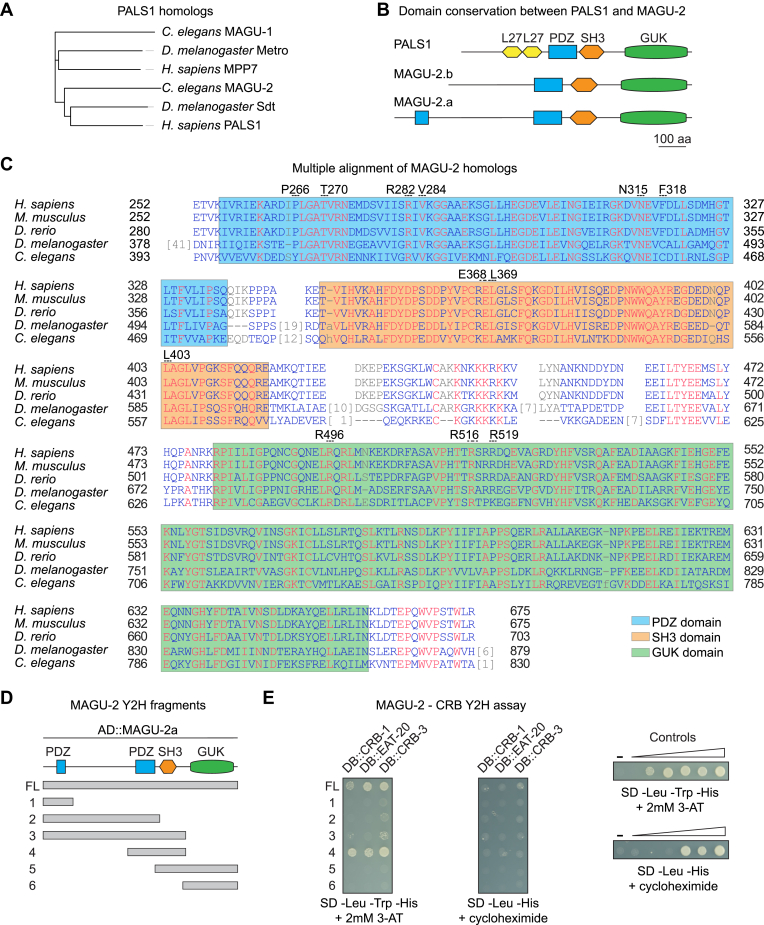
**MAGU-2 is the *Caenorhabditis elegans* ortholog of PALS1.***A*, phylogenetic tree of PALS1 homologs, including *C. elegans* MAGU-1 and -2. *B*, schematic representation of predicted protein domains of PALS1 and MAGU-2. *C*, multiple alignment of MAGU-2a with *Homo sapiens* PALS1, *Mus musculus* Pals1, *Danio rerio* mpp5a, and *Drosophila melanogaster* Sdt. Numbered residues above the sequence are the PALS1 residues involved in the interaction with Crumbs. *D*, schematic representation of the different fragments of MAGU-2 used for the Y2H assay. *E*, interaction of MAGU-2 with CRB-1, EAT-20, and CRB-3 in the Y2H assay. Fragments of MAGU-2b fused to the Gal4 activation domain were coexpressed with the intracellular domain of either CRB-1, EAT-20, or CRB-3 fused to the Gal4 DNA-binding domain. Growth on the -Leu -Trp -His + 2 mM 3-AT plate indicates the presence of interaction. Lack of growth on -Leu -His + cycloheximide plate shows that DB::CRB-1/EAT-20/CRB-3 are not self-activating. Controls range from no reporter activation to strong reporter activation. 3-AT, 3-amino-1,2,4-triazole; GUK, guanylate kinase-like domain; L27, Lin-2 and Lin-7 domain; PDZ, postsynaptic density 95, discs large, zonula occludens-1; SH3 domain, SRC homology 3 domain; Y2H, yeast two hybrid.

To identify candidate PALS1 homologs, we searched the predicted *C. elegans* proteome using BLAST with the human PALS1 and *Drosophila* Sdt sequences. We identified two proteins, MAGU-1 and MAGU-2, that have high sequence similarity, and both contain PDZ, SH3, and GUK domains. However, neither protein appears to harbor the L27 domains present in PALS1. Of the two, MAGU-2 was more closely related to PALS1 and Sdt (Fig. 1*A*). Reciprocal BLAST using MAGU-2 furthermore identified human PALS1 and *Drosophila* Sdt as the closest homologs, while BLAST using MAGU-1 identified human MPP7 and *Drosophila* Metro as the highest scoring hits. MAGU-2 has two predicted isoforms, MAGU-2a of 830 amino acids (aa) and MAGU-2b of 668 aa. A previous analysis of the expression of *magu-2* by rtPCR indicated that *magu-2b* is the predominant isoform (55). Both isoforms consist of a PDZ domain, an SH3 domain, and a GUK domain, while MAGU-2A contains an extra N-terminal PDZ domain which is not present in PALS1 (Fig. 1*B*). The PDZ and SH3 domains of human PALS1 are involved in the interaction with Crumbs, while the GUK domain further enhances the binding. *Drosophila* Crumbs interacts with Sdt through its intracellular PDZ-binding domain (56). Most of the amino acids involved in the interaction between PALS1 and Crumbs are conserved in *C. elegans* MAGU-2 and CRB-1/EAT-20/CRB-3 (Fig. 1*C*). Given the best reciprocal blast, the conservation at the amino acid level, and of the domain structure, we conclude that MAGU-2 is the *C. elegans* ortholog of PALS1.

To determine if MAGU-2 interacts with any of the three *C. elegans* Crumbs proteins, we performed a yeast two hybrid (Y2H) assay. We fused the intracellular domain of the three Crumbs proteins to the DNA-binding domain of Gal4 and fused the following fragments of MAGU-2 to the activation domain: a full-length fragment, an N-terminal fragment with the PDZ present in the long MAGU-2 isoform (frag. 1), an N-terminal fragment including both PDZ domains but lacking the SH3 domain (frag. 2), an N-terminal fragment that includes both PDZ domains and the SH3 domain (frag. 3), a fragment containing the second PDZ domain and the SH3 domain (frag. 4), a C-terminal fragment containing the SH3 and GUK domains (frag. 5), and a C-terminal fragment consisting of only the GUK domain (frag. 6) (Fig. 1*D*). The Y2H assay indicated that full length MAGU-2 interacts with each of the three Crumbs proteins (Fig. 1*E*). Consistent with previous analyses in other organisms (56), both the conserved PDZ domain and the SH3 domain are required for the interaction. These results indicate that the interactions between Crumbs and PALS1 are conserved in *C. elegans*, and that MAGU-2 is a member of the *C. elegans* Crumbs complex.

### MAGU-2 localizes apically in epithelial tissues

**Figure 2:**
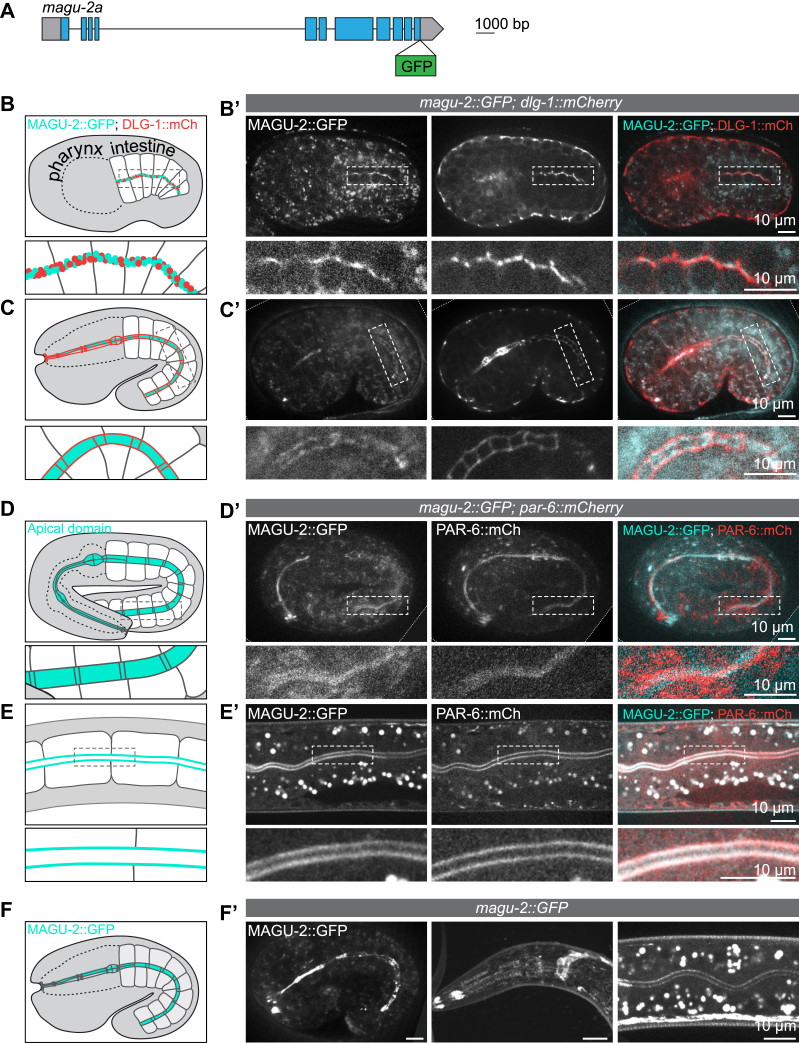
**MAGU-2 localizes apically in epithelial tissues.***A*, schematic representation of endogenous tagging of *magu-2a* with the sequence encoding GFP. *B*–*F*, schematic representations of the areas imaged in *B*′–*F*′, with the localization of MAGU-2 and PAR-6 indicated in *cyan* and the localization of DLG-1 indicated in *red*. *B*′ and *C*′, distribution of MAGU-2::GFP and DLG-1::mCherry in the early stages of intestinal polarization and in comma-shape embryos. *D*′ and *E*′, distribution of MAGU-2::GFP and PAR-6::mCherry in two-fold embryos and L3 larvae. *F*′, distribution of MAGU-2::GFP in the pharynx and intestine of a comma-stage embryo and the nerve ring and intestine of L2/L3 larvae.

To determine a potential role for MAGU-2 in organizing epithelial polarity, we first examined its expression pattern and subcellular localization. Mammalian PALS1 and *Drosophila* Std localize at the apical cortex in epithelial tissues. Thus, if it is a functional ortholog, MAGU-2 would be expected to recapitulate the apical localization of these proteins. To visualize the localization of MAGU-2, we used CRISPR/Cas9 to insert the sequence encoding GFP into the endogenous *magu-2* locus. The tag was inserted at the C-terminus such that both isoforms were tagged (Fig. 2*A*). We first detected MAGU-2::GFP in the intestine during early embryonic development, before apical-basal polarity is fully established, together with DLG-1 at the nascent apical domain (Fig. 2*B*). During later pharyngeal and intestinal development, MAGU-2 localized to the apical cortex, where it colocalized with the apical protein PAR-6 (Fig. 2, *C*, *D*, and *F*). Throughout larval development, MAGU-2::GFP localized to the nerve ring and to the apical membrane domain of the pharynx and intestinal cells (Fig. 2, *E* and *F*). The apical subcellular localization of MAGU-2 in polarized epithelial tissues, which matches the subcellular localization of the Crumbs proteins in these tissues (53, 57), strengthens our hypothesis that MAGU-2 is a functional PALS1 ortholog and a member of the Crumbs complex.

### MAGU-2 is not essential for epithelial polarity

**Figure 3:**
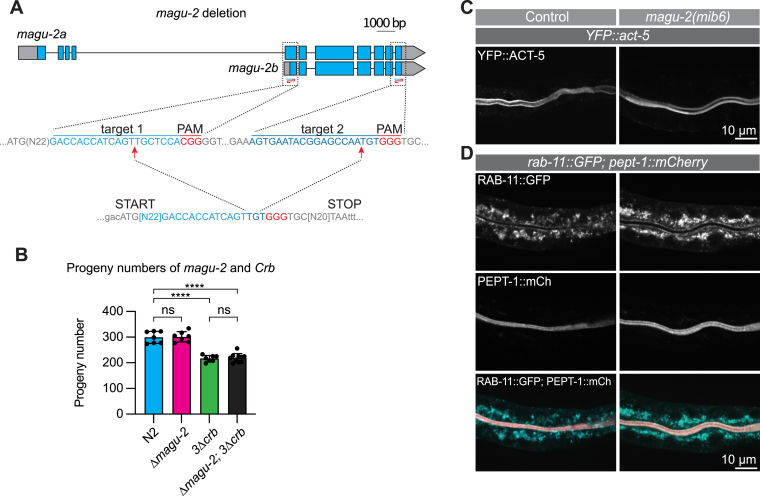
**MAGU-2 is not essential for epithelial polarity.***A*, *top*, genomic *magu-2* gene prediction with sgRNA target sites for the deletion indicated by *blue*/*red* ‘S’ shape symbol. *Gray boxes* indicate untranslated regions. *Middle*, sgRNA target sites for the deletion indicated in *blue text* and PAM sites indicated in *red text*. *Bottom*, sequence of the obtained deletion. *B*, quantifications of brood size. Each *symbol* represents the progeny of an individual animal (*n* = 7, 7, 7, 10). Error bars are the mean ± s.d. Test of significance: Dunnet's multiple comparisons test. ∗∗∗∗*p* ≤ 0.0001. *C*, distribution of YFP::ACT-5 in control or *magu-2(mib6)* deletion larvae. *D*, distribution of RAB-11::GFP and PEPT-1::mCherry in control or *magu-2*(*mib6*) deletion larvae. ns, not significant.

Loss of PALS1/Sdt in mammalian and *Drosophila* epithelia leads to severe polarity defects and disruption of cell junctions (29, 45, 58). The phenotypes of Crumbs and Sdt mutants are very similar, consistent with them forming a core complex (32, 33, 34). To investigate the role of MAGU-2 in polarity establishment, we used CRISPR-Cas9 to generate a *magu-2* deletion allele, *mib6*, that removes 655 out of the 668 or 830 amino acids of the short and long isoforms, respectively, and introduces an early frame shift in the MAGU-2 coding sequence of both isoforms (Fig. 3*A*). Animals homozygous for *magu-2(mib6)* are viable and do not display any obvious developmental defects, suggesting that MAGU-2 activity is dispensable for *C. elegans* development (Fig. 3*B*). These findings are consistent with previous observations that the likely *magu-2* null alleles *ok1059* and *gk218* are viable (55). In order to address any redundancy between MAGU-2 and the Crumbs proteins, we examined the effect of combining the *magu-2* deletion with the triple *crumbs* deletion. We previously reported that a triple *crumbs* deletion results in a small but significant reduction in progeny numbers (53). Combining the *magu-2* deletion with the triple *crumbs* deletion did not exacerbate the phenotype of the *crumbs* deletion mutant (Fig. 3*B*), consistent with both proteins acting in the same pathway.

To assess in more detail whether loss of *magu-2* disrupts apical membrane morphology, we examined the localization pattern of the apically enriched intestine-specific actin isoform ACT-5, the apically enriched recycling endosome marker RAB-11, and the apical membrane transporter PEPT-1 in the absence of *magu-2*. ACT-5 and PEPT-1 localized apically in control animals as well as in *magu-2* deletion mutants (Fig. 3, *C* and *D*). Similarly, RAB-11, which is normally enriched close to the apical lumen in the cytoplasm, maintained the same localization pattern in the absence of MAGU-2 (Fig. 3*D*). Taken together, these data indicate that, like the *crumbs* genes, *magu-2* is not essential for *C. elegans* development and suggest that it may not be necessary for apical polarity.

### Apical localization of MAGU-2 is dependent on Crumbs

**Figure 4:**
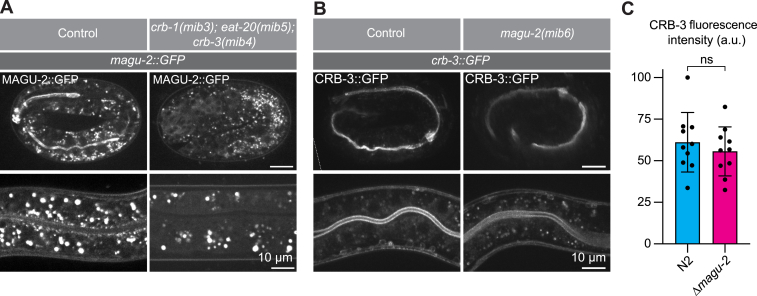
**Subcellular localization of MAGU-2 depends on Crumbs.***A*, distribution of MAGU-2::GFP in control or *crb-1(mib3)*; *eat-20(mib5)*; *crb-3(mib4)* two-fold embryos or larvae. *B*, distribution of CRB-3::GFP in control or *magu-2(mib6)* three-fold embryos or larvae. *C*, quantification of apical CRB-3::GFP fluorescence intensity at the intestinal lumen in control or *magu-2*(*mib6*) deletion larvae. *Individual dots* represent the highest value across the apical domain. The bars represent mean ± SD. n = 5 animals for each condition.

Mammalian PALS1 and *Drosophila* Sdt depend on Crumbs for their apical localization (28, 29, 40). To determine if the apical localization of MAGU-2 similarly depends on the presence of Crumbs proteins in *C. elegans*, we examined the localization of MAGU-2 in the triple *crumbs* deletion mutant. Absence of the three Crumbs proteins resulted in a complete loss of apical accumulation of MAGU-2 in the embryonic and larval intestine (Fig. 4*A*), indicating that Crumbs is necessary for apical enrichment of MAGU-2. In some systems studied, Crumbs apical localization is also dependent on PALS1 or Sdt (14, 29, 31, 45, 59, 60). To determine if this reciprocal requirement is conserved in *C. elegans*, we examined the localization of CRB-3 in the *magu-2(mib6)* deletion strain. To visualize CRB-3, we used a translational CRB-3::GFP fusion expressed from an integrated extrachromosomal array (53). In control animals, CRB-3 localizes to the apical side of the intestine in both embryos and larvae. Upon absence of *magu-2*, the localization of CRB-3 was unaltered (Fig. 3, *B* and *C*), indicating that MAGU-2 does not determine CRB-3 localization in *C. elegans*. Together, these results indicate that the apical recruitment of MAGU-2 depends on Crumbs, while at least the apical localization of CRB-3 is independent of MAGU-2.

### MAGU-2 interacts with the candidate PATJ ortholog MPZ-1

**Figure 5:**
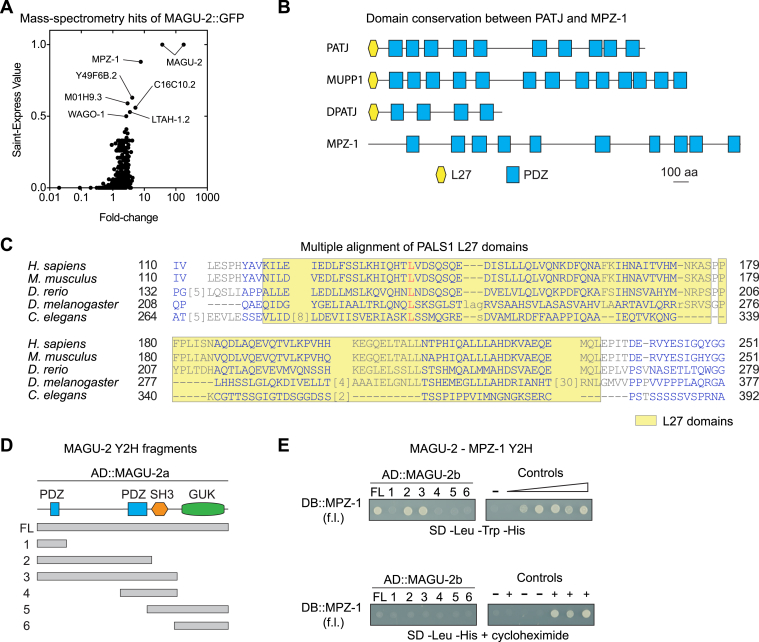
**MAGU-2 interacts with MPZ-1.***A*, mass spectrometry hits for MAGU-2::GFP pull down plotted as correlation between fold-change score and Saint-Express Value. *B*, schematic representation of predicted protein domains of human PATJ, MUPP1, *Drosophila* PATJ, and MPZ-1. *C*, multiple alignment of the L27 domains in *Homo sapiens*, *Mus musculus*, *Danio rerio*, *Drosophila melanogaster*, and *Caenorhabditis elegans*. *D*, schematic representation of the different fragments of MAGU-2 used for the Y2H assay. *E*, interaction of MAGU-2 and MPZ-1 in the Y2H assay. Fragments of MAGU-2b fused to the Gal4 activation domain were coexpressed with the full length of MPZ-1 fused to the Gal4 DNA-binding domain. Growth on the -Leu -Trp -His plate indicates the presence of interaction. Lack of growth on -Leu -His + cycloheximide plate shows that DB::MPZ-1 is not self-activating. Controls are protein pairs that result in known levels of reporter activation and yeast growth, from no reporter activation or growth to strong reporter activation and WT growth levels. L27, Lin-2 and Lin-7 domain; Y2H, yeast two hybrid.

Having established that the *C. elegans* Crumbs complex contains a PALS1 ortholog, we next investigated the existence of a PATJ ortholog. *Drosophila* and mammalian PATJ proteins consist of an L27 domain responsible for the interaction with PALS1 (40) followed by multiple PDZ domains (four in *Drosophila* and ten in mammalian PATJ) (Fig. 5*B*). BLAST searches with the human and *Drosophila* PATJ sequences identified the protein MPZ-1 as the most likely *C. elegans* ortholog. Like mammalian PATJ, MPZ-1 contains ten PDZ domains (Fig. 5*B*). However, MPZ-1 lacks an easily recognizable L27 domain. Moreover, reciprocal BLAST using MPZ-1 identified two possible human orthologs, PALS1 and MUPP1, while the most similar *Drosophila* proteins are the basolateral polarity regulators Dlg and Scrib. Therefore, we explored the interaction partners of MAGU-2 in an unbiased fashion, by performing affinity purification of endogenous MAGU-2::GFP from a mixed-stage population followed by mass-spectrometry. The highest-ranking candidate interacting protein was MPZ-1, further suggesting that MPZ-1 is the *C. elegans* ortholog of PATJ (Fig. 5*A*).

As the L27 domains through which PATJ and PALS1 interact appear to be absent in both MPZ-1 and MAGU-2 (Figs. 1*B* and 5, *B* and *C*), we aimed to identify the MAGU-2 domain responsible for the interaction with MPZ-1. We fused the full length MPZ-1 coding sequence to the DNA-binding domain of Gal4 and examined the interaction with the MAGU-2 activation domain fusions described above using Y2H (Fig. 5*D*). Full length MAGU-2 interacted with MPZ-1, confirming the mass spectrometry result (Fig. 5*E*). MAGU-2 fragments 2 and 3, which contain the region in which the L27 domain is located in mammalian PALS1 and *Drosophila* Sdt, also interacted with MPZ-1. This suggests that the region between the first PDZ domain and the SH3 domain of MAGU-2 is necessary for the interaction between MAGU-2 and MPZ-1, despite lacking a recognizable L27 domain. Taken together, these data indicate that the interactions between Crumbs, PALS1, and PATJ are conserved in *C. elegans*.

**Figure 6:**
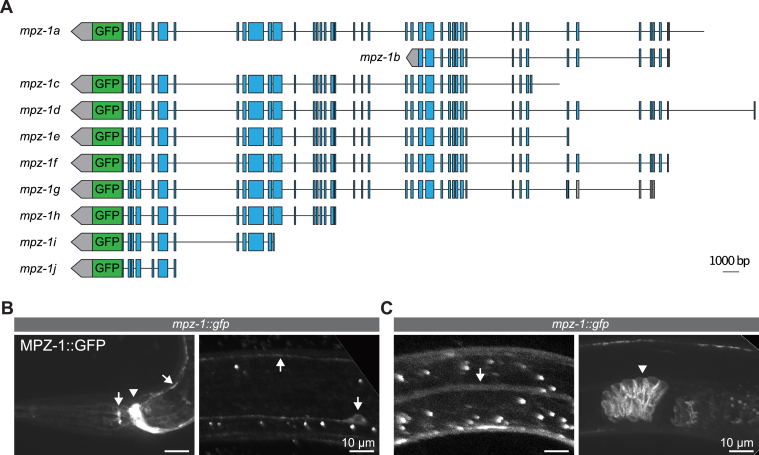
**MPZ-1 accumulates in neurons, the excretory canal, and spermatheca.***A*, schematic representation of endogenous tagging of nine of the ten predicted *mpz-1* isoforms with the sequence encoding GFP. *B*, distribution of MPZ-1::GFP in the nerve ring (*arrowhead*) and neurons (*arrows*). *C*, distribution of MPZ-1::GFP in the excretory canal (*arrow*) and spermatheca (*arrowhead*).

Previous studies identified several candidate roles for MPZ-1, including in serotonin-stimulated egg-laying through an interaction with the serotonin receptor SER-1 and in DAF-2/insulin signaling in a complex with ARR-1^Arrestin^ and DAF-18^PTEN^ (61, 62). Additionally, MPZ-1 was shown to interact with the RhoGEF RHGF-2 and the glutamate receptor MGL-1 (63, 64). Transgene expression studies indicate that MPZ-1 is expressed in the nervous system, body wall muscles, and vulval muscles (62, 63). However, no expression in epithelial tissues has been reported. The *mpz-1* locus is complex, with at least ten predicted splice variants and seven different promoter regions. Hence, previous studies using specific promoters may not have identified the full *mpz-1* expression pattern. We therefore used CRISPR/Cas9 to insert the GFP coding sequences at the 3′-end shared by nine of the ten predicted *mpz-1* isoforms (Fig. 6*A*). Consistent with previous reports (62, 63), MPZ-1 was strongly expressed in the nervous system (Fig. 6*B*). However, we did not observe expression in epithelial tissues, apart from the spermatheca and excretory canal (Fig. 6*C*). Thus, while our data indicate that MPZ-1 interacts with MAGU-2, it is likely not a core member of the epithelial Crumbs complex in *C. elegans*.

### Overexpression of Crumbs causes apical membrane domain expansion

**Figure 7:**
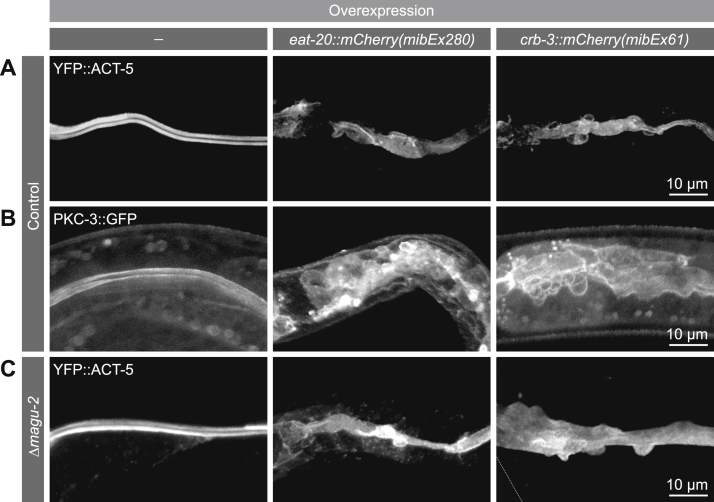
**Crumbs overexpression results in apical domain expansion.***A*, distribution of YFP::ACT-5 in the intestine of larvae in control animals or upon EAT-20 or CRB-3 overexpression. *B*, distribution of PKC-3::GFP in the intestine of larvae in control animals or upon EAT-20 or CRB-3 overexpression. *C*, distribution of YFP::ACT-5 in the intestine of *magu-2*(*mib6*) deletion larvae in control animals or upon EAT-20 or CRB-3 overexpression.

Deletion of all three *crumbs* genes demonstrated that the Crumbs complex is not essential in *C. elegans* (53). However, the effects of Crumbs overexpression, which is known to result in an enlarged apical domain in *Drosophila* (7), have not been investigated in *C. elegans*. In order to induce overexpression, we expressed EAT-20 and CRB-3 with internal mCherry tags under the control of the intestine specific promoter *elt-2* (65). To determine the effect on apical domain size, we examined the distribution of YFP::ACT-5. Expression levels of EAT-20 and CRB-3 from extrachromosomal arrays varied, but in animals expressing the highest levels of either protein, the distribution of ACT-5 indicated an enlarged apical domain, which bulged into the cytoplasm of the intestinal cells (Fig. 7*A*). This indicates that overexpression of EAT-20 or CRB-3 results in apical domain expansion. We next investigated the localization of the endogenously GFP-labeled apical determinant PKC-3 (aPKC) upon EAT-20 or CRB-3 overexpression. Consistent with our interpretation that EAT-20 and CRB-3 overexpression induced an increase in apical domain size, we observed apical localization of PKC-3 in the expanded membrane regions (Fig. 7*B*). Finally, we investigated whether the overexpression phenotype we observed depends on an interaction with MAGU-2. We examined the distribution of YFP::ACT-5 in animals containing the *magu-2(mib6)* deletion allele and overexpressing EAT-20 or CRB-3. Despite the absence of *magu-2*, overexpression of EAT-20 or CRB-3 still induced apical domain expansion (Fig. 7*C*), demonstrating that MAGU-2 is not required for apical membrane expansion.

## Discussion

Here, we characterized the conservation of composition and function of the Crumbs complex in *C. elegans*. We identified MAGU-2 as the *C. elegans* ortholog of mammalian PALS1 and *Drosophila* Sdt. This conclusion is supported by the sequence conservation, the apical localization of MAGU-2, the dependency of MAGU-2 apical localization on Crumbs proteins, and its interaction with each of the Crumbs orthologs. We also identified MPZ-1 as a candidate ortholog of the Crumbs complex component PATJ, based on sequence conservation and the interaction of MAGU-2 with MPZ-1. However, the more restricted expression pattern of MPZ-1, which is not broadly expressed in epithelia, suggests that it is not a general member of the Crumbs complex but may associate with the Crumbs complex in a tissue-specific manner. Finally, we demonstrate that overexpression of the *C. elegans* Crumbs proteins EAT-20 or CRB-3 can induce apical membrane expansion in the intestine, though it does so independently of MAGU-2.

The composition of the Crumbs complex, and the functioning of components besides Crumbs in epithelial polarization, had not been studied in *C. elegans*. Here, we focused on the homolog of PALS1, MAGU-2, which is a core member of the Crumbs complex in mammalian systems and *Drosophila*. Deletion of *magu-2* did not cause any overt defects in epithelial polarization. A previous study found that MAGU-2 regulates phagocytosis in the epidermis to control the number of synapses of cholinergic motor neurons (55). Thus, MAGU-2 may control more specific aspects of the functioning of polarized epithelial cells. MAGU-2 interacted with *C. elegans* CRB-1, EAT-20, and CRB-3 through its PDZ and SH3 domains, as has been shown for Sdt and PALS1 in *Drosophila* and mammals (28, 29, 40). MAGU-2 was enriched at the apical membrane and relied on CRB-1/EAT-20/CRB-3 for its localization. These results are in accordance with previous observations of Sdt and PALS1, both of which localize apically in a Crumbs-dependent manner (28, 29, 40). CRB-3 did not, however, rely on MAGU-2 to become apically enriched. Mammalian Crb3 has been shown to localize apically independently of PALS1 (45), whereas Crb1 has been shown to require PALS1 to localize apically (14). In *Drosophila*, Crumbs has been shown to depend on Sdt in some tissues and developmental stages, such as the embryonic epithelia and mature photoreceptors (29, 31). However in other tissues, such as the posterior midgut and pupal photoreceptors, Crumbs localization is independent of Sdt (59, 60, 66). Thus, the role of PALS1 in localizing Crumbs varies between tissues and organisms. Our efforts here focused on the intestinal localization of CRB-3, hence it remains possible that CRB-1 or EAT-20 depend on MAGU-2 for their apical localization, or that CRB-3 is dependent on MAGU-2 in other tissues.

In addition to the conservation of the interactions with the Crumbs family members, we demonstrated that MAGU-2 interacts with MPZ-1, a candidate ortholog of PATJ. PALS1 and PATJ interact through their L27 domains (39, 40), which do not appear to be conserved in *C. elegans* MAGU-2 and MPZ-1. However, the interaction with MPZ-1 was mediated by the region between the first PDZ domain and SH3 domain of MAGU-2, where the L27 domains are localized in PALS1 proteins in other species. This raises the possibility that MAGU-2 contains an L27-like structural fold that is not recognized at the primary amino acid sequence level. While the sequence conservation and interaction with MAGU-2 suggest that MPZ-1 is an ortholog of PATJ, endogenous tagging of nine out of ten isoforms revealed that most MPZ-1 isoforms are only expressed in the nerve ring and neurons, the excretory canal, and the spermatheca. While we have seen the expression of MAGU-2 and CRB-3 in a small number of neurons, as well as expression of CRB-3 in the excretory canal (53), the expression patterns of these proteins do not overlap to a great extent. This indicates that MPZ-1 is not a member of the core epithelial Crumbs complex but rather a tissue-specific member with more specific functions.

Despite some differences, the *C. elegans* Crumbs complex components are highly conserved at the sequence level, in the interactions between its members, and often in their localization. Nevertheless, none of the *C. elegans* Crumbs complex components have essential roles in the development of *C. elegans* (52, 53, 55). One possible explanation for this difference is that *C. elegans* Crumbs proteins may act redundantly with other proteins. Indeed, CRB-1 was shown to provide a positional cue for junction formation in the intestine upon double depletion of HMP-1 α-catenin and LET-413 Scribble (54), suggesting that redundant mechanisms ensure proper junction formation in the intestine. Further supporting a role for the *C. elegans* Crumbs proteins in regulating apical-basal polarization, and similarly to what has been observed in *Drosophila* (7), we observed that overexpression of either EAT-20 or CRB-3 resulted in apical membrane expansion. This phenotype did not depend on the presence of MAGU-2. While we cannot exclude indirect effects of overexpressing EAT-20 or CRB-3, it is possible that the apical expansion is mediated by interactions with alternative binding partners. Two candidates for such partners are the apical polarity determinant PAR-6 and the membrane–actin linker ERM-1, homologs of which have been shown to directly interact with Crumbs proteins in other systems and mediate aspects of Crumbs functioning (21, 67, 68, 69). Thus, the precise function of the Crumbs proteins in *C. elegans* remains elusive.

Redundancy in the polarity network has been seen in other systems, such as *Drosophila*, where Crumbs is expressed in all embryonic epithelia derived from the ectoderm but tissues react differently to loss of Crumbs, from disintegration and apoptosis in the epidermis to no apparent defects in the hindgut (70, 71). These findings highlight that in order to understand the different functions of polarity regulators, it is important to study them in a range of different systems and organisms. Although it remains uncertain what the precise role is in *C. elegans* of the Crumbs complex, our characterization provides further insight into the biology of these evolutionarily conserved components in polarized epithelia.

## Experimental procedures

### Homology searches

Homology searches were performed using BLAST (72) with the input sequences of human PALS1 (Q8N3R9-1) and PatJ (Q9NB04), *Drosophila* Sdt (M9NG38-1) and PatJ-PC (Q8NI35) and *C. elegans* MAGU-2 (C01B7.4b.1), MAGU-1 (Q95XW5-1) and MPZ-1 (G5ECZ8-1). The following sequences were obtained from UniProt (73) and used for the homology tree: MAGU-1 (Q95XW5), MAGU-2 (Q17549), PALS1 (Q8N3R9), and Sdt (Q8WRS3), MPP7 (Q5T2T1) and Metro (A1Z8G0). The homology tree was constructed using Clustal Omega (74) and “Tree of Life” (https://itol.embl.de/itol.cgi) (75). The following sequences were obtained from UniProt and used for determining the domain conservation between PatJ and MPZ-1: MPZ-1 (G5ECZ8), PATJ (Q8NI35), MUPP1 (O75970), and *Drosophila* PATJ (Q9NB04).

### Y2H analysis

Sequences encoding MAGU-2, CRB-1, EAT-20, CRB-3, and MPZ-1 were PCR amplified from a mixed-stage cDNA library using the primers in Table 1. PCR products were digested with AscI and NotI and cloned into Gal4-DB vector pMB28 and Gal4-AD vector pMB29, respectively (76). The resulting plasmids were transformed into *Saccharomyces cerevisiae* strains Y8930 (MATα) and Y8800 (MATa) using the Te/LiAc transformation method (77). Diploid yeast was generated by mating and plated on synthetic defined medium plates (1) lacking leucine, tryptophan, and histidine and containing 2 mM 3-amino-1,2,4-triazole to assess the presence of an interaction while reducing background growth; (2) lacking leucine, tryptophan, and adenine to assess the presence of an interaction and (3) on a synthetic medium plate lacking leucine and histidine and containing 1 μg/ml cycloheximide to test for self-activation. Controls of known reporter activation strength and behavior on cycloheximide were also added to all plates.

**Table 1:** Primers used for yeast two-hybrid assay

Primer	Sequence
oVGC342_mpz1_Y2H_FL_F	GGAGGCGCGCCATGCCGTTACAATCCGAGGA
oVGC343_mpz1_Y2H_FL_R	GGAGCGGCCGCTCATTGTTGAGGGATACTGT
oVGC348_magu2_Y2H_FL_F	GGAGGCGCGCCATGTCCAAGTCGGTGTCGAT
oVGC349_magu2_Y2H_FL_R	GGAGCGGCCGCTTAGCACGCTGTCCATGTAG
oVGC350_magu2_Y2H_1_R	GGAGCGGCCGCTTAATTCGAATTTGTGCAGTTAA
oVGC351_magu2_Y2H_2_R	GGAGCGGCCGCTTAAGACTTCTTTGCTGTGACAG
oVGC352_magu2_Y2H_3_R	GGAGCGGCCGCTTATGGAAGATTTTCCTCGTCAG
oVGC353_magu2_Y2H_4_F	GGAGGCGCGCCATGTCCCCCATCCCACCAGTTAT
oVGC354_magu2_Y2H_5_F	GGAGGCGCGCCATGGAACAAGACACCGAACAACC
oVGC355_magu2_Y2H_6_F	GGAGGCGCGCCATGGAGGTCAAAAAAGGGGCTGA
oVGC356_crb1_Y2H_intra_F	GGAGGCGCGCCATGCGGGGCAATAACGCCATGCA
oVGC357_crb1_Y2H_intra_R	GGAGCGGCCGCTCAGATAAGACGTTCTTGAGG
oVGC358_eat20_Y2H_intra_F	GGAGGCGCGCCATGTACATTCGCCAGTCACGTAA
oVGC359_eat20_Y2H_intra_R	GGAGCGGCCGCTTAGATCAGCCGCTCCTCCT
oVGC360_crb3_Y2H_intra_F	GGAGGCGCGCCATGAAATATGTGAAAGATAGACGAAAAAACC
oVGC361_crb3_Y2H_intra_R	GGAGCGGCCGCTTAGATAAGTCCTTCTACATTCGG

### *C. elegans* strains

All *C. elegans* strains used in this study are derived from the N2 Bristol strain and are listed in Table 2. All strains were maintained at 20 °C on Nematode Growth Medium (NGM) plates seeded with *Escherichiae coli* OP50 bacteria under standard conditions (78). Table 2 contains a list of all strains used.

**Table 2:** Strain list

Strain	Genotype	Acknowledgment
JM125	*caIs108[Pges-1p::YFP::ACT-5]*	J. McGhee
BOX301	*magu-2(magu-2::GFP::loxP) V; dlg-1(mib23) X*	
BOX300	*par-6(mib25[par-6::mCherry-LoxP]) I; magu-2(mib36) V*	
BOX267	*magu-2(magu-2::GFP::loxP) V*	
BOX42	*mibIs24[crb-3::GFP- Avi, Pmyo-3::mCherry] IV*	
BOX161	*magu-2(mib6) V*	
BOX164	*magu-2(mib6) V; crb-1(mib3), eat-20(mib5), crb-3(mib4) X*	
BOX176	*magu-2(mib6) V; mibIs24[crb-3::GFP-2TEV-Avi; Pmyo-3::mCherry] IV*	
BOX198	*magu-2(mib6) V; caIs[Pges-1::YFP::ACT-5]*	
MZE1	*unc-119(ed3) III; cbgIs91[pPept-1:pept-1::DsRed;unc-119(+)]; cbgIs98[pPept-1:GFP::rab-11.1;unc-119(+)]*	M. Zerial
BOX182	*magu-2(mib6) V; unc-119(ed3) III; cbgIs91[pPept-1:pept-1::DsRed;unc-119(+)]; cbgIs98[pPept-1:GFP::rab-11.1;unc-119(+)]*	
BOX283	*magu-2(magu-2::GFP::loxP) V; crb-1(mib3), eat-20(mib5), crb-3(mib4) X*	
BOX64	*mibIs39[Prps-27::GFP-2xTEV-Avi 10 ng/μl + Prab-3::mCherry 5 ng/μl + lambda DNA 65 ng/μl] I*	
BOX443	*pkc-3(mib78[egfp-loxp::aid::pkc-3]) II*	
pD1074	*C. elegans* WT isolate	
BOX866	*mpz-1(mib188[mpz-1::gfp]) II*	

### CRISPR/Cas9 genome engineering

The *magu-2::GFP* fusion was done by homology-directed repair of CRISPR/Cas9-induced DNA double-strand breaks in an N2 background. The fusion was generated using plasmid-based expression of Cas9 and sgRNAs. The sequence targeted by the sgRNA was 5′ GTGAATACGGAGCCAATGT. The *magu-2::GFP* repair template was cloned using SapTrap assembly into vector pDD379, and the fusion was repaired using a plasmid-based template with 550 to 600 bp homology arms. A self-excising cassette (SEC) was used for selection (79). The homology arms included mutations of the sgRNA recognition site to prevent recutting after repair. The injection mix was prepared in MilliQ H_2_O and contained 50 ng/μl Peft-3::cas9 (Addgene ID #46168) (80), 65 ng/μl sgRNA-repair-template vector, and 2.5 ng/μl coinjection pharyngeal marker Pmyo-2::tdTomato to aid in visual selection of transgenic strains. Young adult hermaphrodites were injected in the germline using an inverted micro-injection setup (Eppendorf FemtoJet 4× mounted on a Zeiss Axio Observer A.1 equipped with an Eppendorf Transferman 4r). Candidate-edited progeny were selected on plates containing 250 ng/μl of hygromycin (79), and correct genome editing was confirmed by PCR amplification of the edited genomic region. From correctly edited strains, the hygromycin selection cassette was excised by heat shock of L1 larvae at 34 °C for 1 h in a water bath. Correct excision was confirmed by Sanger sequencing (Macrogen Europe) of PCR amplicons encompassing the edited genomic region.

The *magu-2(mib6)* deletion was generated by imprecise repair of a CRISPR/Cas9-induced DNA double-strand breaks and selected using the *dpy-10* co-CRISPR approach (81). The fusion was generated using plasmid-based expression of Cas9 and sgRNAs. Two pairs of sgRNA plasmids were used to target the 5′ and 3′ ends of the *magu-2* open reading frame and generated by ligation of annealed oligo pairs into the *pU6::sgRNA* expression vector pMB70 (Addgene #47942) as previously described (82). The sequences targeted by the sgRNA were as follows: 5′ GAGTACATTGCTGCAACATC; 5′ GACCACCATCAGTTGCTCCA; 5′ AGTGAATACGG AGCCAATGT; and 5′ AATTCTAATGAAAGTGAATA. The sgRNA targeting the *dpy-10* locus (81) was cloned into the *pU6::sgRNA* expression vector pJJR50 (Addgene #75026) as previously described (83). The injection mix was prepared in MilliQ H_2_O and contained 60 ng/ml *Peft-3::Cas9* (Addgene #46168), 45 ng/ml each sgRNA plasmid, and 2.5 ng/ml *Pmyo-2::mCherry* as a coinjection marker (pCFJ90, Addgene #19327). Microinjection of adult N2 hermaphrodites was performed as described above. For selection of edited genomes, injected animals were transferred to individual culture plates, incubated for 3 to 4 days at 20 °C, and 96 nontransgenic F1 animals (WT, Dpy, or Rol) from 2 to 3 plates containing high numbers of Dpy and Rol animals were selected and transferred to individual NGM plates. After laying eggs, F1 animals were lysed and genotyped by PCR using two primers flanking the *magu-2* open reading frame: 5′ to 3′ left primer CATACGCCCAA TCATCCGCAC, right primer GATGATGATGGTGTCTC TTCTG. Sanger sequencing was used to determine the precise molecular lesion (Macrogen Europe), and the confirmed knockout strain was backcrossed three times with N2.

The *mpz-1(mib188)* allele was generated by homology-directed repair of CRISPR/Cas9-induced DNA double-strand breaks in an N2 background (strain pD1074). The fusion was generated using protein-based expression of Cas9 and crRNAs. Two crRNA were used, targeting 5′ CATTGTTGAGGGATACTGTG and 5′ tactagtctttTCATTGTTG. The GFP repair template was generated by PCR from pJRK86 using the following 5′Sp9-modified primers: 5′ TTCAAATTGCCCGTC CACACAGTATCCCTCAACAAATGAGTAAAGGAGAAG AATT and 5′ tgtgtgagagagggtctacagtactagtctttTCAGTAGAG CTCGTCCATTCCGT. The injection mix contained 30 pmol *Streptococcus pyogenes* Cas9 3NLS, 90 pmol tracrRNA, 47.5 pmol of each crRNA, 25 ng/μl melted dsDNA, 40 ng/μl of pRF4 *(rol-6(su1006))* plasmid, and nuclease-free water to bring the final volume to 20 μl (84). Young adult hermaphrodites were injected in the germline using an inverted micro-injection setup (Eppendorf FemtoJet 4× mounted on a Zeiss Axio Observer A.1 equipped with an Eppendorf Transferman 4r). Candidate edited progeny were selected from the plates containing roller progeny, and correct genome editing was confirmed by PCR amplification of the edited genomic region using primers 5′ caagcttgagccctgctagt and 5′ tttaagggggaa gcggtacg. Correct insertion was confirmed by Sanger sequencing (Macrogen Europe) of PCR amplicons encompassing the edited genomic region.

### Brood size

Starting at the L4 stage, individual P0 animals were cultured at 20 °C and transferred to a fresh plate every 24 h for 6 days. For each plate, hatched F1 progeny was scored 24 h after removal of the P0.

### Microscopy

Live imaging of *C. elegans* larvae was done by mounting larvae on 5% agarose pads in a 10 mM Tetramisole solution in M9 buffer to induce paralysis. Spinning disk confocal imaging was performed using a Nikon Ti-U microscope driven by MetaMorph Microscopy Automation & Image Analysis Software (Molecular Devices) and equipped with a Yokogawa CSU-X1-M1 confocal head and an Andor iXon DU-885 camera, using 60× or 100× 1.4 NA objectives. All stacks along the z-axis were obtained at 0.25 μm intervals, and all images were analyzed and processed using ImageJ (FIJI) and Adobe Photoshop.

### Quantitative image analysis

Image analysis was done in ImageJ (FIJI). The peak intensity at the apical membrane was measured by drawing a ten pixels wide line scan from the cytoplasm, across the apical membrane and to the lumen. The highest value of the line scan across the apical membrane was selected and the mean background intensity, quantified on a circular region in an area not containing any animals, was subtracted from the intensity measurements. All measurements were taken from regions where opposing apical membranes could be seen clearly as two lines and two intestinal cells per animal were quantified.

### GFP pull-down of MAGU-2::GFP

Animals endogenously expressing GFP-tagged MAGU-2 or control animals expressing an integrated GFP transgene (83) were grown on 6 to 8 9-cm NGM plates until starvation, to enrich for L1 animals. Animals were then transferred into 250 ml of S-Medium supplemented with 1% Penn/Strep (Life Technologies), 0.1% nystatin (Sigma), and OP50 bacteria obtained from the growth of a 400 ml culture. Animals were grown at 20 °C at low shaking for 96 h and were harvested and cleaned using a sucrose gradient, as previously described (83) with one exception being the inclusion of MgSO4 in the M9 medium. Worms were distributed into 15 ml TPX tubes (Diagenode) to reach 200 to 400 μl worm pellet per tube and washed with lysis buffer (25 mM Tris–HCl pH 7.5, 150 mM NaCl, 1 mM EDTA, 0.5% IGEPAL CA-630, 1X Complete Protease Inhibitor Cocktail (Roche)). The liquid was removed, and the sample was flash frozen in liquid nitrogen for storage at −80 °C. To lyse the worms, tubes were thawed on ice and ice-cold lysis buffer was added to reach a total volume of 2 ml. Tubes were sonicated for 10 min (sonication cycle: 30 s ON, 30 s OFF) at 4 °C in a Bioruptor ultrasonication bath (Diagenode) at high energy setting. After lysis, lysates were cleared by centrifugation and protein levels were measured using the Bradford bicinchoninic acid assay (Thermo Scientific). Immunoprecipitation was performed using GFP-Trap Magnetic Agarose beads (Chromotek) according to manufacturer’s protocol, using 25 μl of beads per sample. To prep the beads, they were first equilibrated in wash buffer (10 mM Tris/Cl pH 7.5, 150 mM NaCl, 0.5 mM EDTA, 0.1% IGEPAL CA-630), blocked with 1% bovine serum albumin for 1 h, and then washed 4 times with wash buffer. Next, lysate was added to the beads and they were incubated for 1 h tumbling end-over-end. Lysate was then removed, and the beads were washed four times in wash buffer. After the final wash step, all liquid was removed, and the beads were flash frozen with liquid nitrogen. The experiment was performed in triplicate (biological replicates) and processed on independent days.

### Mass spectrometry analysis for MAGU-2

After affinity purification, anti-GFP beads were resuspended in 15 μl of 4× Laemmli sample buffer (Biorad), boiled at 99 °C for 10 min, and the supernatants were loaded on 4 to 12% Criterion XT Bis–Tris precast gel (Biorad). The gel was fixed with 40% methanol and 10% acetic acid and then stained for 1 h using colloidal Coomassie dye G-250 (Gel Code Blue Stain, Thermo Scientific). Each lane from the gel was cut and placed in 1.5 ml tubes. Samples were then washed with 250 μl of water, followed by 15 min dehydration in acetonitrile. Proteins were reduced (10 mM DTT, 1 h at 56 °C), dehydrated, and alkylated (55 mM iodoacetamide, 1 h in the dark). After two rounds of dehydration, trypsin was added to the samples and incubated overnight at 37 °C. Peptides were extracted with acetonitrile, dried down, and reconstituted in 10% formic acid prior to mass spectrometry analysis.

Samples were analyzed on an Orbitrap Q-Exactive mass spectrometer (Thermo Fisher Scientific) coupled to an Agilent 1290 Infinity LC (Agilent Technologies). Peptides were loaded onto a trap column (Reprosil pur C18, Dr Maisch, 100 μm × 2 cm, 3 μm; constructed in-house) with solvent A (0.1% formic acid in water) at a maximum pressure of 800 bar and chromatographically separated over the analytical column (Poroshell 120 EC C18, Agilent Technologies, 100 μm × 50 cm, 2.7 μm) using 90 min linear gradient from 7% to 30% solvent B (0.1% formic acid in acetonitrile) at a flow rate of 150 nl min^−1^. The mass spectrometers were used in a data-dependent mode, which automatically switched between MS and MS/MS. After a survey scan from 375 to 1600 m/z, the ten most abundant peptides were subjected to HCD fragmentation. MS spectra were acquired with a resolution >30,000, whereas MS2 with a resolution >17,500.

Raw data files were converted to mgf files using Proteome Discoverer 1.4 software (Thermo Fisher Scientific). Database search was performed using the *C. elegans* database and Mascot (version 2.5.1, Matrix Science) as the search engine. Carbamidomethylation of cysteines was set as a fixed modification and oxidation of methionine was set as a variable modification. Trypsin was set as cleavage specificity, allowing a maximum of two missed cleavages. Data filtering was performed using a percolator, resulting in 1% false discovery rate. Additional filters were search engine rank 1 and mascot ion score >20. Source of sequence database searched are as follows: Uniprot, release date: October 24, 2017 (27,546 total entries). Mass tolerance for precursor ions was 50 ppm and mass tolerance for fragment ions was 0.05 Da.

Crapome (85) was used to analyze MAGU-2 interacting proteins in three biological replicas, using proteins identified in the GFP pull downs, as well as in pull downs of GFP::PAR-3 (BOX290), GFP::PKC-3 (KK1228), and DLG-1::GFP (BOX260), as controls. Significance analysis of interactome score and simpler fold-change (FC) calculations FC-A and FC-B were derived from the Crapome analysis by averaging the spectral counts across the controls. FC-A averages the counts across all controls while the more stringent FC-B takes the average of the top three highest spectral counts for the abundance estimate.

### Overexpression of *eat-20* and *crb-3*

The *pelt-2::eat-20::mCherry* construct was cloned into the pBSK(+) vector using Gibson assembly. The promoter of *elt-2*, which drives expression in the intestine, was amplified from *C. elegans* genomic DNA using primers 5′ TAATTTCGAAATGTATGAACTCCA and 5′ CTATAATCTATTT TCTAGTTTCTATTTTATTAGA. A coding fragment of 777 bp including introns was amplified from genomic DNA using primers 5′ ATGACCACGTTTTGTCGAGT and 5′ TCGATTGGATACGGATCGAA. A fragment of 1817 bp without introns was amplified from cDNA using primers 5′ TCACGGGACCAGCCTGCCTA and 5′ GATGTGAGACATCGGCA TCG. A fragment of 749 bp containing the 3′ of *eat-20* and 725 bp of 5′ UTR was amplified from genomic DNA using primers 5′ GCCAAGGAGGAGCGGCTGAT and 5′ tagaattttattaattatta. mCherry was amplified from pJJR83 (Addgene #75028) using primers 5′ TCCAAGGGAGAGG AGGACAA and 5′ GTAGAGCTCGTCCATTCCTC. Correct amplification and assembly were confirmed by Sanger sequencing.

The *pelt-2::crb-3::mCherry* construct was cloned into the pMLS257 vector using SapTrap assembly (86). The promoter of *elt-2* was amplified from *C. elegans* genomic DNA using primers 5′ CTGCTCTTCgTGGTAATTTCGAAATGTATGA AC and 5′ GTGCTCTTCgGTCCTATAATCTATTTTCTA GTT. A fragment of 886 bp containing introns was amplified from genomic DNA using primers 5′ GGCTGCTCTTCgGACATGGCGTCAAACAGTACGGT and 5′ GGGTGCTCTTC gCATAGGTTGAAGATATGGTAGGT. A fragment of 200 bp containing the 3′ of *crb-3* and 176 bp of 5′ UTR was amplified from genomic DNA using primers 5′ GGCTGCTC TTCgAAGCCGAATGTAGAAGGACTTAT and 5′ GGGTG CTCTTCg TACgcggataagtttta-tttttgtac. The assembly was performed using 100 fmol of each fragment, 100 fmol of FP:mCherry (PMB 78), and 100 fmol of the destination vector (pMLS257). Correct amplification and assembly were confirmed by Sanger sequencing.

To generate transgenic lines, young adult hermaphrodites were injected in the germline with the following mix: 65 ng/μl of plasmid of interest, 2.5 ng/μl of *pmyo-2::TdTomato*, and 50 ng/μl of lambda DNA. GFP fluorescence was used to select stable transgenic lines.

## Data availability

The mass spectrometry proteomics data have been deposited to the ProteomeXchange Consortium (87) *via* the PRIDE partner repository (88) with the dataset identifier PXD030122 and are included in Table S1. For strain requests, please contact the corresponding author.

## Supporting information

This article contains supporting information.

## Conflict of interest

The authors declare that they have no conflicts of interest with the contents of this article.

## Supporting information

Supplemental Table S1 Excel file containing the mass spectrometry data. The first sheet contains the Crapome analysis of MAGU-2::GFP, GFP::PAR-3, GFP::PKC-3, and DLG-1::GFP separately against a cytoplasmic GFP control. The second sheet contains the analysis of MAGU-2 that uses the combined interaction profiles of cytoplasmic GFP, GFP::PAR-3, GFP::PKC-3, and DLG-1::GFP to identify MAGU-2::GFP specific interactors, as plotted in Figure 5.
